# Multicomponent reactions IV

**DOI:** 10.3762/bjoc.21.163

**Published:** 2025-10-14

**Authors:** Thomas J J Müller, Valentyn A Chebanov

**Affiliations:** 1 Heinrich-Heine-Universität Düsseldorf, Institut für Organische Chemie und Makromolekulare Chemie, Universitätsstraße 1, D-40225 Düsseldorf, Germany; 2 Institute of Functional Materials Chemistry, State Scientific Institution “Institute for Single Crystals” of National Academy of Sciences of Ukraine, Nauky Av. 60, 61072 Kharkiv, Ukraine; 3 Faculty of Chemistry, V. N. Karazin Kharkiv National University, Svobody Sq. 4, 61077 Kharkiv, Ukraine

**Keywords:** multicomponent reactions

The synthesis of complex molecules is a cornerstone of modern science. Chemistry is not only concerned with understanding and modifying chemical bonds but also with creating enabling technologies that help to address challenges at the interface of materials science and the life sciences. The growing need to provide substance libraries for the identification and optimization of promising lead structures has therefore inspired the development of powerful synthetic strategies. A glance at Web of Science reveals that one-pot methods and multicomponent reactions (MCRs) [1] have attracted steadily increasing attention within the scientific community over the past 25 years (Figure 1).

**Figure 1:**
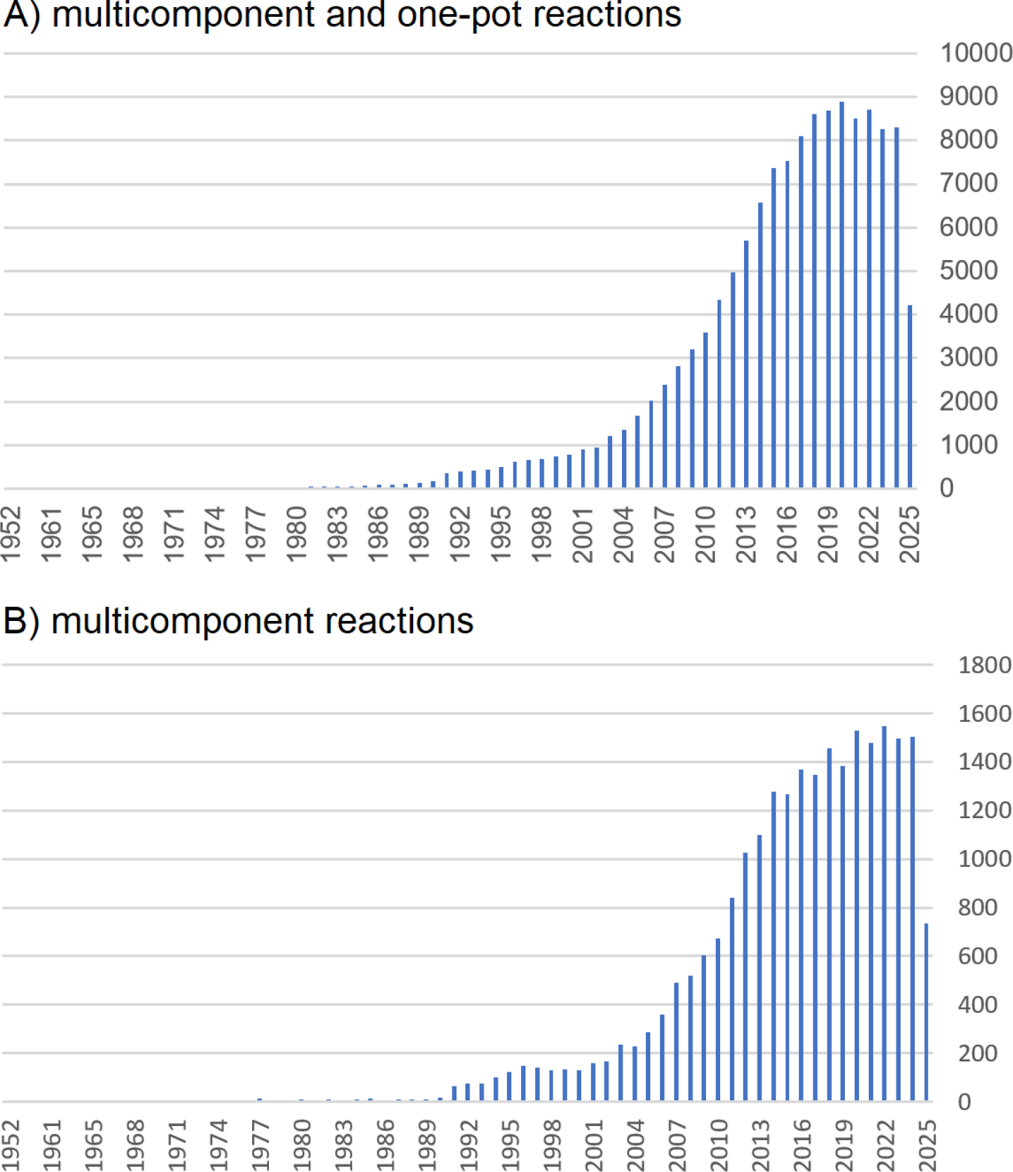
Number of publications by year on multicomponent and one-pot reactions (A) and multicomponent reactions alone (B) accessed by Web of Science on June 16, 2025.

According to a quick Web of Science [2] keyword search of "multicomponent reaction" and "one-pot reaction" (Figure 1A) and "multicomponent reaction" (Figure 1B) filtered by publication year, the impressive sum of 136,658 publications on multicomponent (Figure 1A) and one-pot reactions and 24,369 publications on MCRs alone (Figure 1B) clearly underline the central importance and topicality of the MCR concept. In sensu stricto, MCRs involve the reaction of three or more components in a single vessel, proceeding under domino, sequential, or consecutive control without the need for intermediate purification, work-up, or solvent exchange. Domino processes [3] are characterized by the simultaneous presence of all reactants from the outset, whereas sequential reactions permit the controlled addition of components while maintaining the same reaction conditions. Consecutive processes, in turn, even allow changes in conditions from one step to the next. In essence, MCRs embody a reactivity-driven concept [4] within the broader family of one-pot methodologies.

This thematic issue on multicomponent reactions continues the previously published editions from 2011 [5], 2014 [6], and 2019 [7] and is guest-edited by Thomas J. J. Müller (Heinrich Heine University Düsseldorf, Germany) together with Valentyn A. Chebanov (State Scientific Institution “Institute for Single Crystals” of the National Academy of Sciences of Ukraine, Kharkiv, Ukraine). The 30 contributions collected in this thematic issue (1 Letter, 22 Full Research Papers, and 7 Reviews) reflect the broad spectrum of MCR chemistry worldwide.

Among the Full Research Papers, isocyanide-based multicomponent reactions still constitute the relative majority. Ugi and co-workers’ groundbreaking discovery of the four-component reaction between an aldehyde, an amine, a carboxylic acid, and an isonitrile in 1959 [8], which marked the beginning of modern MCR chemistry, continues to attract undiminished attention. It has since been applied in manifold ways, from breathtaking reaction sequences and post-Ugi transformations to the generation of countless molecules, spanning small compounds to microstructures. The amphiphilic reactivity of isonitriles appears virtually limitless, extending well beyond classical Ugi-type reactions into other MCR contexts. At the same time, MCRs not based on isonitriles are gaining increasing momentum, with a strong emphasis on heterocycle synthesis. Beyond traditional condensation-based approaches, mechanistically innovative crossovers – linking metal catalysis with radical chemistry and, more recently, with photo(redox) catalysis – are opening entirely new avenues for MCR development. Finally, seven focused Reviews on heterocycles, reactivity patterns, and the role of MCRs as enabling tools at the interface with biosciences underscore the timeliness and enduring elegance of this powerful and concise concept in molecular synthesis.

As the guest editors of this thematic issue, we would like to thank all the authors – dedicated and outstanding scientists – for sharing their exciting findings. We are especially grateful to the staff of the Beilstein-Institut for their excellent support and professional execution of this project.

Thomas J. J. Müller and Valentyn A. Chebanov

Düsseldorf and Kharkiv, September 2025

## Data Availability

Data sharing is not applicable as no new data was generated or analyzed in this study.
